# SGLT2 inhibitors and GLP-1 receptor agonists: impact on mortality in diabetic patients with cardiovascular disease

**DOI:** 10.1186/s12933-025-02874-7

**Published:** 2025-08-31

**Authors:** Ziad Arow, Tzipi Hornik-Lurie, Ranin Hilu, Ela Giladi, Yoav Arnson, Hana Vaknin-Assa, Abid Assali, David Pereg, For the CARDIAB research group

**Affiliations:** 1 Cardiology Department, Meir Medical Center, 59 Tchernichovsky St, 44281 Kfar Saba, Israel; 2 Gray Faculty of Medical and Health Sciences, Tel-Aviv University, Tel-Aviv, Israel; 3 Meir Medical Center Research Institute, Kfar Saba, Israel

**Keywords:** Type 2 diabetes mellitus, Atherosclerotic cardiovascular disease, Sodium-glucose cotransporter 2 inhibitors (SGLT2-I), Glucagon-like peptide-1 receptor agonists (GLP-1RA)

## Abstract

**Background:**

Sodium-glucose cotransporter 2 inhibitors (SGLT2-I) and glucagon-like peptide-1 receptor agonists (GLP-1RA) have been shown to reduce cardiovascular risk and mortality in patients with type 2 diabetes mellitus (T2D), yet remain underutilized in clinical practice. This study aimed to evaluate real-world treatment patterns and associated mortality outcomes among patients with T2D and established atherosclerotic cardiovascular disease (ASCVD).

**Methods:**

The CARdiovascular and DIABetes (CARDIAB) cohort included 138,397 patients with T2D and ASCVD. Patients were categorized into four treatment groups: (i) both SGLT2-I and GLP-1RA, (ii) SGLT2-I only, (iii) GLP-1RA only, and (iv) neither medication. The primary outcome was all-cause mortality.

**Results:**

Of the 138,397 patients, 57% received neither SGLT2-I nor GLP-1RA, 17% received both, 20% received SGLT2-I only, and 6% received GLP-1RA only. Female sex, older age, non-coronary ASCVD, and absence of follow-up in specialized cardiology or diabetes clinics were associated with lower treatment rates. Compared to those receiving neither medication, all-cause mortality was significantly lower among patients treated with SGLT2-I only (HR 0.28, 95% CI 0.27–0.29), GLP-1RA only (HR 0.39, 95% CI 0.37–0.40) and both agents (HR 0.17, 95% CI 0.16–0.18). This association remained significant following a multivariate analysis.

**Conclusion:**

In patients with T2D and ASCVD, treatment with SGLT2-I and GLP-1RA, especially in combination, is associated with a substantial reduction in mortality. These findings highlight significant gaps in implementation and the urgent need to optimize use of evidence-based therapies in this high-risk population.

## Research insights

**What is currently known about this topic?**

Sodium-glucose cotransporter 2 inhibitors (SGLT2-I) and glucagon-like peptide-1 receptor agonists (GLP-1RA) have been shown to reduce cardiovascular risk and mortality in patients with type 2 diabetes mellitus (T2D) and established cardiovascular disease, yet remain underutilized in clinical practice.

**What is the key research question?**

What are the real-world treatment patterns and associated mortality outcomes among patients with T2D and established atherosclerotic cardiovascular disease (ASCVD)?

**What is new?**

- Using data from the large real-world CARdiovascular and DIABetes (CARDIAB) cohort, we identified a marked underutilization of SGLT2 inhibitors and GLP-1 receptor agonists among patients with type 2 diabetes and established cardiovascular disease. Lower treatment rates were particularly evident among women, older individuals, patients with non-coronary atherosclerotic cardiovascular disease, and those lacking follow-up in specialized cardiology or diabetes clinics.
- Both SGLT2 inhibitors and GLP-1 receptor agonists were associated with a significant survival benefit, with the greatest effect observed in patients treated with combination therapy.

**How might this study influence clinical practice?**

- Our study highlights a pressing need for healthcare systems and policymakers to develop and implement strategies that enhance the adoption of these guideline-directed therapies.
- Our findings support the consideration of dual therapy in appropriately selected high-risk patients.

## Introduction

Patients with type 2 diabetes mellitus (T2D) and established atherosclerotic cardiovascular disease (ASCVD) face a substantially elevated risk of major adverse cardiovascular events and premature mortality [1–3]. In recent years, two classes of glucose-lowering medications,sodium-glucose cotransporter 2 inhibitors (SGLT2-I) and glucagon-like peptide-1 receptor agonists (GLP-1RA),have emerged as cornerstone therapies due to their robust cardiovascular and renal protective effects. Large randomized clinical trials have consistently demonstrated that these agents not only improve glycemic control but also significantly reduce rates of cardiovascular death, heart failure hospitalization, and progression of chronic kidney disease [4–6]. Reflecting this evidence, major international guidelines, now provide class I recommendations for the use of SGLT2-I and GLP-1RA in patients with T2D and high cardiovascular risk,regardless of baseline glycemic control or HbA1c levels [7, 8]. Despite these strong recommendations, real-world studies indicate that SGLT2-I and GLP-1RA remain markedly underutilized in routine practice [9–11]. This therapeutic gap is concerning, as observational data suggest that failure to initiate these agents is associated with increased morbidity and mortality [12]. The factors contributing to this underutilization are multifactorial and may include demographic disparities, clinical complexity, healthcare access, provider awareness, and socioeconomic barriers.

The present study aimed to evaluate treatment patterns of SGLT2-I and GLP-1RA in a large cohort of patients with T2D and concomitant ASCVD identify predictors of their underuse, and examine the association between these therapies and all-cause mortality in real-world clinical practice.

## Methods

### Study population

The CARdiovascular and DIABetes (CARDIAB) cohort study was conducted using the electronic health records database of Clalit Health Services (CHS), the largest integrated payer-provider healthcare system in Israel, serving over 4.5 million members—approximately 55% of the Israeli population. The CHS database contains comprehensive information on patient demographics, clinical characteristics, diagnoses from hospital and outpatient clinics, medical treatments, medication dispensation, and laboratory test results. Data extraction was performed via the Clalit research data-sharing platform powered by MDClone (https://www.mdclone.com). Clinical diagnoses were identified using International Classification of Diseases, Ninth Revision (ICD-9) codes.

The cohort included patients aged > 18 years with T2D and established ASCVD, defined as coronary artery disease (acute myocardial infarction or coronary revascularization), cerebrovascular accident (CVA) or transient ischemic attack (TIA), or peripheral arterial disease (PAD). Patients with end-stage kidney disease or those treated with SGLT2-I or GLP-1RA prior to the first ASCVD diagnosis were excluded. The study period spanned from January 1, 2019, to December 31, 2024.

The study population was categorized into four groups: (i) patients treated with both GLP-1RA and SGLT2-I; (ii) patients treated with SGLT2-I only; (iii) patients treated with GLP-1RA only; and (iv) patients not treated with either GLP-1RA or SGLT2-I. Treatment with GLP-1RA or SGLT2-I was defined as at least three dispensations of the medication. Baseline characteristics and clinical outcomes were compared across the four groups. The primary outcome was all-cause mortality. Mortality rates were assessed by cross-referencing patient identification numbers with the Israeli National Population Registry.

The study was approved by the local institutional ethics committee in accordance with the principles of the Declaration of Helsinki. In line with Ministry of Health regulations, written informed consent was not required, as data were anonymized and collected retrospectively from electronic medical records without direct patient involvement.

### Statistical analysis

*Descriptive statistics and group comparisons*: Baseline characteristics were summarized using descriptive statistics. Continuous variables were presented as mean with standard deviation, and categorical variables were presented as frequencies and percentages.

Between-group comparisons of baseline characteristics were performed using appropriate statistical tests. For continuous variables, the Wilcoxon rank-sum test was used. For categorical variables, Pearson's chi-square test was used when expected cell frequencies were ≥ 5 in at least 80% of cells; otherwise, Fisher’s exact test was employed.

*Incidence analysis and Poisson regression*: Incidence rates were calculated as the number of events per person-time at risk, expressed per 10,000 person-years. Incidence rate ratios (IRRs) with 95% confidence intervals were estimated using Poisson regression models with robust variance estimation to account for potential overdispersion. The exposure time was included as an offset variable in the models.

*Time-to-event analysis*: Kaplan–Meier survival curves were constructed to estimate event-free survival probabilities by study group. Patients were followed from the index date until the occurrence of the cardiac outcome, death, or end of follow-up, whichever occurred first. The index event was defined as either the initial clinical presentation ASCVD encompassing coronary, cerebrovascular, or peripheral arterial disease in a patient with pre-existing diabetes, or the first diagnosis of diabetes in a patient with established ASCVD.

*Cox proportional hazards models*: Cox proportional hazards regression was used to estimate hazard ratios (HRs) and 95% confidence intervals for the association between study group and time to outcome. The proportional hazards assumption was assessed using weighted residuals. Multivariable Cox models were fitted, adjusting for pre-specified baseline covariates identified as clinically important or showing imbalance between groups (SMD > 0.10) in univariable analyses. In addition to the study group, covariates included age group, sex, cardiovascular risk factors, comorbidities and other relevant prognostic factors.

*Statistical software and significance*: All analyses were performed using R software (version 4.2.3). Two-sided *p*-values < 0.05 were considered statistically significant.

## Results

The CARDIAB cohort comprised 138,397 patients with T2D and concomitant ASCVD, with a median follow-up of 65 (30.1, 72.5) months. Overall, the use of guideline-recommended therapies was suboptimal: 78,611 patients (57%) were not treated with either a GLP-1RA or a SGLT2-I. In contrast, 23,897 patients (17%) received both medications, 8,507 (6%) were treated with GLP-1RA only, and 27,382 (20%) received SGLT2-I only. Baseline demographic and clinical characteristics stratified by treatment group are summarized in Table 1. Characteristics associated with lower rates of GLP-1RA or SGLT2-I use included older age, female sex, chronic kidney disease (CKD), and non-cardiac vascular diseases such as cerebrovascular accident/transient ischemic attack (CVA/TIA) and peripheral vascular disease (PVD). As anticipated, GLP-1RA use was more prevalent among patients with obesity. In contrast, follow-up in cardiology and diabetes specialty clinics was positively associated with the likelihood of receiving either or both medication classes (Table 2).

**Table 1:** Baseline characteristics according to treatment group

Treatment group	SGLT2-I and GLP-1RA	GLP-1RA	SGLT2-I	No SGLT2-I or GLP-1RA	*p*-value
n	23,897	8507	27,382	78,611	
Age, years (median ± SD)	65 ± 9	68 ± 10	70 ± 10	76 ± 11	*p* < 0.001
*Age Group, n (%)*					
19–60	6806 (29)	2021 (24)	4509 (17)	7296 (9%)	*p* < 0.001
61–74	13,787 (58)	4271 (50)	14,097 (52)	25,583 (32)	*p* < 0.001
75 +	3304 (14)	2215 (26)	8776 (32)	45,732 (58)	*p* < 0.001
Gender, male n (%)	16,255 (68)	4275 (50)	19,515 (71)	42,524 (54)	*p* < 0.001
Obesity, n (%)	19,271 (80)	7230 (85)	14,640 (54)	45,442 (58)	*p* < 0.001
BMI (median ± SD)	32 ± 5	33 ± 6	28 ± 5	29 ± 5	*p* < 0.001
Dyslipidemia, n (%)	17,895 (75)	6083 (72)	19,437 (71)	53,936 (69)	*p* < 0.001
Hypertension, n (%)	21,489 (90)	7626 (90)	23,697 (87)	70,821 (90)	*p* < 0.001
CKD, n (%)	2763 (11)	1618 (19)	2961 (11)	16,781 (21)	*p* < 0.001
Smokers, n (%)	13,846 (58)	4225 (50)	14,959 (55)	34,793 (45)	*p* < 0.001
*Cardiovascular disease*					
MI, n (%)	10,999 (46)	2669 (31)	13,885 (51)	30,359 (39)	*p* < 0.001
CVA/TIA, n (%)	6095 (26)	3228 (38)	7482 (27)	34,524 (44)	*p* < 0.001
PVD, n (%)	2957 (12)	1253 (15)	3358 (12)	12,097 (15)	*p* < 0.001
PCI, n (%)	11,820 (49)	3010 (35)	13,104 (49)	27,188 (35)	*p* < 0.001
Heart failure, n (%)	5805 (24)	1929 (23)	7552 (27)	23,893 (30)	*p* < 0.001
*Medications*					
Statins	23,242 (97)	8093 (95)	26,315 (96)	73,500 (93)	*p* < 0.001
ACE-I/ARB	21,579 (90)	7311 (86)	23,941 (87)	67,583 (86)	*p* < 0.001
Beta-blockers	19, 510 (82)	6408 (75)	21,835 (80)	61,679 (78)	*p* < 0.001
Aspirin	22,719 (95)	7820 (92)	25,463 (93)	71,853 (91)	*p* < 0.001
Other anti-diabetes drugs	23,354 (97)	8062 (95)	25,570 (93)	65,070 (83)	*p* < 0.001

SGLT2-I, sodium-glucose cotransporter 2 inhibitor; GLP-1RA, Glucagon-like peptide-1 receptor agonist; BMI, body mass index; CKD, chronic kidney disease; MI, myocardial infarction; CVA, cerebrovascular accident; TIA, transient ischemic attack; PVD, peripheral vascular disease; PCI, percutaneous coronary intervention; ACE-I, angiotensin converting enzyme inhibitor; ARB, angiotensin receptor blocker

**Table 2:** Treatment rates of SGLT2-I and GLP-1RA according to follow-up care

Treatment group	SGLT2-I and GLP-1RA	GLP-1RA	SGLT2-I		No SGLT2-I or GLP-1RA	*P*-value
n (138,397)	23,897	8507	27,382		78,611	
*Follow-up clinic*						
Cardiology and Diabetes, n (%)	5624 (24)	1475 (17)	4448 (16)	5380 (7)		*P* < 0.001
Cardiology only, n (%)	12,538 (53)	3824 (45)	15,485 (57)	27,699 (35)		*P* < 0.001
Diabetes only, n (%)	1294 (6)	570 (7)	1073 (4)	2618 (3)		*P* < 0.001
None, n (%)	4441 (18)	2620 (31)	6376 (23)	42,914 (55)		*P* < 0.001

SGLT2-I, sodium-glucose cotransporter 2 inhibitor; GLP-1RA, Glucagon-like peptide-1 receptor agonist

**Fig. 1:**
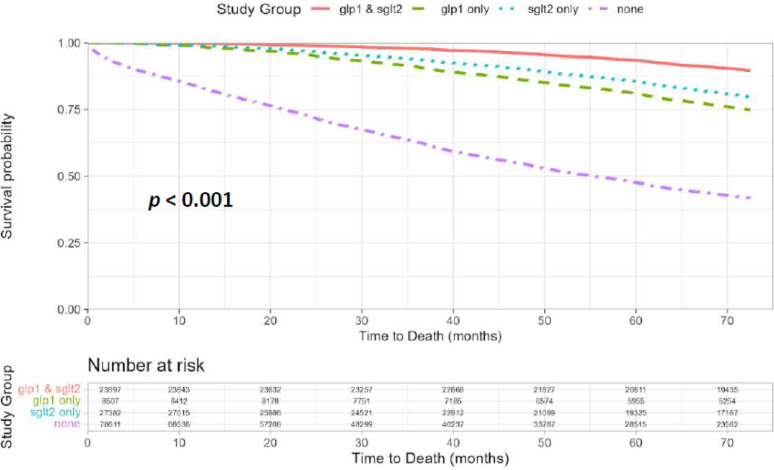
Kaplan–Meier survival curves according to treatment group

**Fig. 2:**
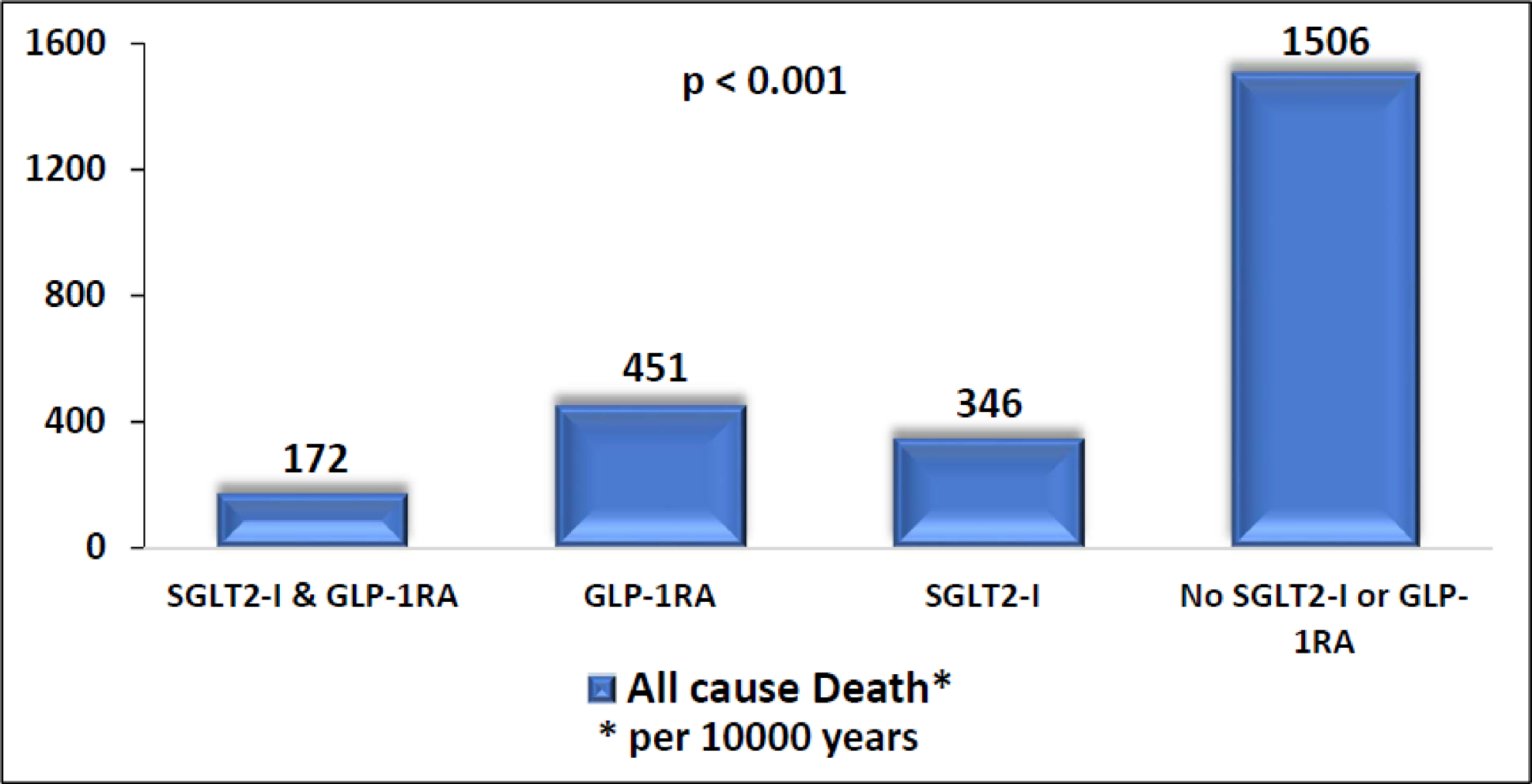
All cause death per 10,000 patient years according to treatment group

Treatment with SGLT2-I and GLP-1RA was significantly associated with improved survival (Fig. 1), with the highest survival rates observed in patients treated with both medication classes. The all-cause mortality rates per 10,000 patient-years were 172 for those treated with both medications, 346 for SGLT2-I only, 451 for GLP-1RA only, and 1506 for those not treated with either (*p* < 0.001) (Fig. 2). These associations remained significant following a multivariate analysis (Table 3). Using the group not treated with either medication as the reference, the hazard ratios (HR) for all-cause mortality were: HR = 0.28 (95% CI 0.26–0.28) for SGLT2-I only, HR = 0.38 (95% CI 0.36–0.4) for GLP-1RA only and HR = 0.17 (95% CI 0.16–0.17) for both medications. Combination therapy with both SGLT2-I and GLP-1RA was associated with significantly lower all-cause mortality compared to monotherapy with SGLT2-I (HR = 0.61, 95% CI 0.58–0.64) or GLP-1RA (HR = 0.44, 95% CI 0.35–0.47).

**Table 3:** Cox regression model—all cause death

Characteristic	HR^1^	95% CI^1^	*p*-value
*Study group*			
None		—	
GLP-1RA & SGLT2-I	0.17	0.16, 0.18	<.0001
GLP-1RA only	0.39	0.37, 0.4	<.0001
SGLT2-I only	0.28	0.27, 0.29	<.0001
*Gender*			
Female		—	
Male	1.01	0.99, 1.03	0.3859
*Age group*			
19–60		—	
61–74	1.7	1.62, 1.77	<.0001
75 +	3.29	3.15, 3.43	<.0001
*Follow up clinic*			
Cardiology	0.82	0.80, 0.84	<.0001
Cardiology and diabetes	0.83	0.80, 0.85	<.0001
Smoking	1.1	1.08, 1.12	<.0001
Prior MI	1.36	1.33, 1.39	<.0001
Obesity	0.99	0.97, 1.01	0.1291
Prior TIA/CVA	1.33	1.31, 1.36	<.0001
PVD	1.35	1.32, 1.38	<.0001
Hypertension	1.16	1.11, 1.2	<.0001
CKD	1.59	1.56, 1.62	<.0001
Hyperlipidemia	0.98	0.95, 0.99	0.0198
Statins	0.83	0.79, 0.87	<.0001
Aspirin	0.89	0.86, 0.93	<.0001
Beta blockers	1.1	1.07, 1.13	<.0001
ACE-I/ ARB	1.15	1.11, 1.19	<.0001
Other anti-diabetes drugs	1.17	1.14, 1.2	<.0001

^1^HR, hazard ratio, CI, confidence interval

SGLT2-I, sodium-glucose cotransporter 2 inhibitor; GLP-1RA, glucagon-like peptide-1 receptor agonist; CKD, chronic kidney disease; MI, myocardial infarction; CVA, cerebrovascular accident; TIA, transient ischemic attack; PVD, peripheral vascular disease; ACE-I, angiotensin converting enzyme inhibitor; ARB, angiotensin receptor blocker

## Discussion

*The CARDIAB study* aimed to evaluate real-world treatment patterns and outcomes among patients with diabetes and concomitant cardiovascular disease. In this large, nationwide cohort of 138,397 patients, we found that despite strong clinical guideline recommendations, the use of SGLT2-I and GLP-1RA remains low. Factors independently associated with lower prescription rates included older age, female sex, CKD, and the presence of non-coronary cardiovascular disease. In contrast, referral to specialized cardiology or diabetes clinics was linked to higher treatment rates. Lack of treatment with SGLT2i or GLP-1RA was associated with the highest risk of all-cause mortality.

The cardiovascular benefits of treatment with either SGLT2-I or GLP-1RA in high-risk patients with type 2 diabetes (T2D) have been well established in multiple randomized controlled trials [13–16]. However, evidence supporting the combined use of both drug classes is primarily derived from retrospective studies [12, 17–19]. A large retrospective cohort analysis involving patients with T2D receiving insulin, across 85 healthcare organizations, evaluated the effect of SGLT2-I, GLP-1RA, and combination therapy on clinical outcomes [12]. Compared to a propensity-matched control group, each treatment approach was associated with reduced all-cause mortality and improved cardiovascular outcomes, with the greatest benefit observed in the combination therapy group. Another large retrospective study, analysing data from over 400,000 U.S. adults with T2D, found that combination therapy with SGLT2-I and GLP-1RA resulted in significantly better cardiometabolic outcomes, and reduced risk of stroke and myocardial infarction, compared with SGLT2-I alone [17]. In a smaller propensity score–matched study of patients with T2D and established coronary artery disease, 208 matched pairs were compared between those treated with SGLT2-I alone versus combination therapy with GLP-1RA [18]. The combined use of both agents was associated with a significantly reduced risk of cardiovascular mortality and stroke. Additionally, a prospective study of 443 patients post–acute myocardial infarction evaluated outcomes of treatment with either SGLT2-I, GLP-1RA, or both [19]. Patients in good glycemic control maintained the same glucose-lowering regimen while those with HbA1C > 7% were prescribed either SGLT-2I or GLP-1RA to obtain a combination therapy. The incidence of major adverse cardiovascular events (MACE) was significantly lower in the combination group compared to those receiving monotherapy. The current study adds to the growing body of evidence by providing a comprehensive, real-world assessment of treatment patterns and outcomes among high-risk patients with T2D and established cardiovascular disease. First, it includes a large, unselected cohort representative of routine clinical practice. Second, it evaluates treatment rates and predictors of underuse of SGLT2-I and GLP-1RA. Finally, it demonstrates a significant survival benefit associated with both drug classes.

Our findings carry several important clinical implications. First, the marked underutilization of both SGLT2 inhibitors and GLP-1 receptor agonists, despite their well-documented benefits, and the associated reduction in survival underscore the urgent need for healthcare systems and policymakers to implement strategies that promote broader use of these therapies. Targeted efforts should be directed toward populations with the lowest treatment rates, including women and older adults. Second, we observed significantly higher prescription rates of SGLT2-I and GLP-1RA among patients followed in cardiology or diabetes specialty clinics. This highlights the pivotal role of specialized care in optimizing the management of high-risk patients with T2D and suggests that expanding access to such clinics may improve adherence to evidence-based therapies. Finally, our results add to the growing body of evidence supporting the superiority of combination therapy with SGLT2-I and GLP-1RA over either monotherapy.

This study has several limitations that warrant consideration. First, as a retrospective observational analysis, the cohort is subject to healthy subject treatment bias. Patients who were prescribed SGLT2-I or GLP-1RA may represent a subgroup that is generally healthier, more health-conscious, or more engaged with the healthcare system. These individuals may possess other favorable characteristics, beyond the use of SGLT2-I or GLP-1RA, that could independently contribute to improved outcomes. Although we adjusted for a wide range of baseline clinical and demographic characteristics, the possibility of residual confounding by unmeasured factors, such as lifestyle, frailty, or socioeconomic status, remains. Second, the database lacked information on specific causes of death, limiting the analysis to all-cause mortality as the primary endpoint. Third, Treatment with GLP-1RA or SGLT2-I was defined as at least three dispensations of the medication. Unfortunately, our database did not include information regarding treatment duration, distribution of time from index date to treatment initiation, the percentage of covered time or whether both medications were taken concurrently in patients receiving combination therapy. Fourth, Although initial clinical evidence supporting the cardiovascular benefits of SGLT2-I and GLP-1 RA in high-risk diabetic patients emerged in 2015, several additional large-scale trials were published in the following years. These accumulating data led to progressive updates and strengthening of international clinical guideline recommendations for the use of SGLT2-I and GLP-1 RA therapies throughout the study period. Finally, while the CARDIAB cohort represents a large, national real-world population, the generalizability of these findings to other healthcare systems or countries should be approached with caution.

*In conclusion*, among patients with T2D and concomitant cardiovascular disease, treatment with SGLT2-I and GLP-1 RA is associated with a significant survival benefit, with the greatest effect observed in those receiving combination therapy. Despite this, real-world treatment rates with these medications remain suboptimal, highlighting a critical gap in the implementation of evidence-based care.

## Data Availability

Availability of data and materials: The datasets used and/or analysed during the current study are available from the corresponding author on reasonable request.

## Abbreviations

T2D: Type 2 diabetes mellitus
ASCVD: Atherosclerotic cardiovascular disease
SGLT2-I: Sodium-glucose cotransporter 2 inhibitors
GLP-1RA: Glucagon-like peptide-1 receptor agonists
CARDIAB: CARdiovascular and DIABetes
CHS: Clalit health services
CVA: Cerebrovascular accident
TIA: Transient ischemic attack
PAD: Peripheral arterial disease
CKD: Chronic kidney disease
